# Treatment with silver nitrate versus topical steroid treatment for umbilical granuloma: A non-inferiority randomized control trial

**DOI:** 10.1371/journal.pone.0192688

**Published:** 2018-02-13

**Authors:** Chikako Ogawa, Yoshiaki Sato, Chiyo Suzuki, Azusa Mano, Atsushi Tashiro, Takafumi Niwa, Sayako Hamazaki, Yoshihiro Tanahashi, Midori Suzumura, Satoshi Hayano, Masahiro Hayakawa, Takeshi Tsuji, Shin Hoshino, Yuichiro Sugiyama, Hiroyuki Kidokoro, Jun-ichi Kawada, Hideki Muramatsu, Akihiro Hirakawa, Masahiko Ando, Jun Natsume, Seiji Kojima

**Affiliations:** 1 Department of Pediatrics, Nagoya University Graduate School of Medicine, Nagoya, Japan; 2 Division of Neonatology, Center for Maternal-Neonatal Care, Nagoya University Hospital, Nagoya, Japan; 3 Department of Pediatrics, Okazaki City Hospital, Okazaki, Japan; 4 Department of Pediatrics, Kasugai City Hospital, Kasugai, Japan; 5 Department of Biostatistics and Bioinformatics, Graduate School of Medicine, The University of Tokyo, Tokyo, Japan; 6 Center for Advanced Medicine and Clinical Research, Nagoya University Hospital, Nagoya, Japan; 7 Brain and Mind Research Center, Nagoya University, Nagoya, Japan; 8 Department of Developmental Disability Medicine, Nagoya University Graduate School of Medicine, Nagoya, Japan; National Cancer Institute, UNITED STATES

## Abstract

**Objective:**

The aim of this prospective multicenter randomized controlled trial was to compare the efficacy of silver nitrate cauterization against that of topical steroid ointment in the treatment of neonatal umbilical granuloma.

**Methods:**

An open-label, non-inferiority randomized controlled trial was conducted from January 2013 to January 2016. The primary endpoint for the silver nitrate cauterization and topical steroid ointment groups was the healing rate after 2 weeks of treatment, applying a non-inferiority margin of 10%. The healing rate was evaluated until completion of 3 weeks of treatment.

**Results:**

Participants comprised 207 neonates with newly diagnosed umbilical granuloma, randomized to receive silver nitrate cauterization (n = 104) or topical steroid ointment (n = 103). Healing rates after 2 weeks of treatment were 87.5% (91/104) in the silver nitrate cauterization and 82% (82/100) in the topical steroid ointment group group. The difference between groups was -5.5% (95% confidence interval, -19.1%, 8.4%), indicating that the non-inferiority criterion was not met. After 3 weeks of treatment, the healing rate with topical steroid ointment treatment was almost identical to that of silver nitrate cauterization (94/104 [90.4%] vs. 91/100 [91.0%]; 0.6% [-13.2 to 14.3]). No major complications occurred in either group.

**Conclusions:**

This study did not establish non-inferiority of topical steroid ointment treatment relative to silver nitrate cauterization, presumably due to lower healing rates than expected leading to an underpowered trial. However, considering that silver nitrate cauterization carries a distinct risk of chemical burns and that the overall efficacy of topical steroid ointment treatment is similar to that of silver nitrate cauterization, topical steroid ointment might be considered as a good alternative in the treatment of neonatal umbilical granuloma due to its safety and simplicity. To clarify non-inferiority, a larger study is needed.

## Introduction

Umbilical granuloma (UG) is the most common umbilical abnormality in neonates. The granulation tissue in the lesion is soft, 3–10 mm in size, vascular, dull red or pink in color, and may show a seropurulent secretion [1].

The most commonly used treatment for UG is silver nitrate cauterization (SNC) [1, 2]. However, chemical burns to the periumbilical area, eyelids, and so on caused by SNC have been reported as possible complications [3–6]. Various studies have investigated alternatives to SNC, including common salt [7], cryosurgery or electrocautery [8], excision and application of absorbable hemostatic materials [9], double ligatures [10], and alcoholic wipes [5]. Topical steroid ointment (TSO) treatment for UG has gained wide attention due to the advantages of effectiveness and simplicity [11, 12]. Brodsgaard et al raised the possibility that topical clobetasol propionate (0.05%), as a group IV steroid, may be as effective as SNC for treatment of UG [11]. However, that study was not large enough to show superiority or non-inferiority to SNC.

Since every primary-care physician may encounter neonates with UG, clearer evidence of benefit is essential before advocating TSO treatment. We therefore conducted a prospective multicenter randomized controlled trial (RCT) of TSO treatment for UG. The aim of this study was to examine the non-inferiority of TSO to SNC in neonates with UG.

## Materials and methods

This prospective, nonblinded, multicenter RCT compared SNC and TSO treatments in neonates with newly diagnosed UG. The trial was approved by the institutional review board of every participating hospital: Ethics Review Committee of Nagoya University Graduate School of Medicine, which approved the trial for Nagoya University Hospital, Narita Hospital and Clinic Mama; the institutional review board (IRB) of Anjo Kosei Hospital; the IRB of Ogaki Municipal Hospital; the ethics committee of Okazaki City Hospital; the Chubu Rosai Hospital Research Ethics Committee; the IRB of Tsushima City Hospital; the IRB of Nishichita General Hospital; Rinri-kojinjyohohogokanriiinkai of Toyota Memorial Hospital; the Toyota Kosei Hospital Institutional Review Board; the Nakatsugawa Municipal General Hospital ethics committee; the IRB of Nagoya Memorial Hospital; the ethics committee of Japanese Red Cross Nagoya Daiichi Hospital; the Research Ethics Committee of Japan Post Nagoya-teishin Hospital; the IRB of Tosei General Hospital; the Research Ethics Committee of Japanese Community Health Care Organization Chukyo Hospital; the IRB of Handa City Hospital; the IRB of Kasugai City Hospital; and the Clinical Research Review Committee of Konan Kosei Hospital. Written informed consent was obtained from the parents of each neonate prior to enrollment. The trial was conducted in accordance with the ethical standards laid down in the 1964 Declaration of Helsinki.

**Fig 1:**
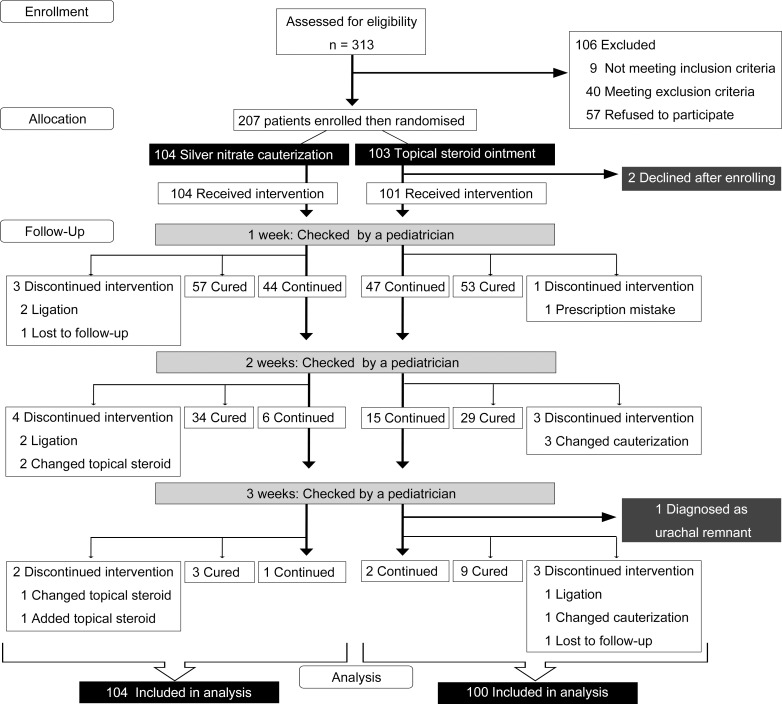
CONSORT flow diagram. Progression of patients through the trial up to 3 weeks of treatment in all participating centers. No patients dropped out due to adverse events in either group.

Patients were 207 neonates with UG who were consecutively followed at 18 hospitals and 2 clinics in Japan from January 2013 to January 2016. Inclusion criteria were: 1) age, 1–5 weeks; and 2) diagnosis of UG from clinical features (soft, round, wet, often pink, often pedunculated lesion ≥3 mm in diameter in the umbilicus) [1, 2], made by experienced pediatricians in each hospital or clinic. Exclusion criteria were: 1) infection in the umbilicus; 2) prior treatment of UG; 3) prior systemic antibiotic treatment within 1 week; or 4) prior surgical treatment of the umbilicus. All patients meeting the inclusion criteria were randomized to receive either SNC or TSO treatment (Fig 1).

We randomly assigned patients in a 1:1 ratio to receive treatment with TSO or SNC. The independent data center of Nagoya University Hospital performed the randomization using web-based software that generates a random number and includes a minimization procedure by prognostic factors (sex: male or female; age at diagnosis: <15 days old, between 15 and 29 days old, or ≥30 days old; gestational age: <34 weeks, between 34 and 36 weeks, or ≥37 weeks; birth body weight: <2000 g, between 2000 and 2999 g, or ≥3000 g). No blinding was applied in the present study, since it was impossible for physicians/parents to perform either treatment in a blinded manner. Telephone and online services were available at all times for assignment of patients.

### Clinical trial registry name and registration number

This trial was registered to the University Hospital Medical Information Network Clinical Trials Registry (UMIN-CTR) (http://www.umin.ac.jp).

Registry name: A prospective multicenter randomized controlled study of the efficacy of betamethasone valerate on umbilical granuloma.

Registration number: UMIN000009397

### SNC

Patients were treated once a week by pediatricians using silver nitrate applied using a clean stick applicator at a concentration of 20% in a hospital or clinic. The stick was dried before application, the surrounding skin was protected with petroleum jelly and contact with normal skin was avoided. Prior to treatment, we informed the parents about the following possible side effects: 1) chemical burns in the periumbilical area; and 2) pigmentation changes in the cauterized area. Parents were advised to avoid normal baths for the baby for 24 h in order to prevent the SCN from causing periumbilical burns.

### TSO treatment

Patients were applied with 0.05% betamethasone valerate ointment to the lesion twice a day after washing or bathing by parents. No occlusive dressing was used and no attempt was made to dry the lesion. Prior to treatment, we informed the parents about the following possible side effects: 1) local infection at the umbilicus; and 2) stinging, pigmentation, itching, irritation or skin-color changes. Parents were explained to avoid occlusive dressings in order to avoid enhancement of drug absorption.

### Follow-up

All patients attended the outpatient clinic once a week. Patients were followed-up until the lesion was judged to be healed. The same treatment was repeated 1 week later if the lesion was not healed. In a previous study that retrospectively compared SNC and TSO treatment in children with UG [12], mean healing times for SNC (n = 12) and TSO treatment (n = 16) were 12.0 and 10.3 days, respectively. We therefore allowed each pediatrician to change or add another treatment after 2 weeks of the initially allocated treatment.

### Endpoints and definitions

The trial primary endpoint was the healing rate for the SNC and TSO groups after 2 weeks of treatment. The secondary endpoint was the time to healing.

Healing was defined as no clinical sign of umbilical exudate, bleeding or granulation as judged by experienced pediatricians. Treatment failure was defined as persistence of granulation tissue at the base of the umbilicus that was large enough for each pediatrician to consider ligation, changing or addition of another treatment after 2 weeks of treatment, or occurrence of adverse clinical events.

### Measurements

Demographic data included birth age, birth weight, age at enrollment, gender and underlying disease. After enrollment, the presence or absence of any of umbilical exudate, bleeding and granulation was recorded every week until healing was achieved.

All data were collected and entered into databases by the pediatricians who diagnosed and/or followed-up UG at each outpatient clinic.

### Statistical analysis

In the sample size calculation for this non-inferiority trial, we assumed that the healing rate would be 95% for both the SNC and TSO treatment groups. The non-inferiority margin was set at 10%; that is, the aim of this non-inferiority trial was to demonstrate that TSO treatment was no more than 10% worse than SNC with respect to the healing rate. To this end, a sample size of 100 patients per group was required to achieve more than 80% power with a two-sided type 1 error of 5%. Analysis was undertaken on an intention-to-treat basis. All patients who were randomized and received any treatment were eligible for inclusion in the primary efficacy analysis. Participants who had no data on the primary endpoint, did not satisfy all inclusion criteria or satisfied any exclusion criteria, or did not receive any treatment were excluded from the primary analysis. The primary efficacy analysis compared healing rates and 95% confidence intervals (CIs) based on the profile likelihood method [13] between groups. Distributions of baseline characteristics were compared using Fisher’s exact tests and t tests.

## Results

A flow diagram of this trial is shown in Fig 1. A total of 207 patients met the inclusion criteria and were randomized to each intervention group. As treatment effects could not be evaluated in several patients in the present study, indicated that we could not evaluate the treatment effect, 7 more patients were registered. One hundred and four patients were allocated to the SNC group, and 103 were allocated the TSO treatment group. Of these 207 patients, 204 patients completed the trial, including 104 patients (100%) treated with SNC and 100 patients (97%) treated with TSO. Three patients were withdrawn, comprising 2 infants whose parents declined further participation after enrollment, and 1 infant who was diagnosed with urachal remnant. At the 4-week checkup, 1 patient who continued with SNC and 1 patient who continued with TSO treatment achieved cure, and one patient who continued with TSO treatment was changed to SNC. The main baseline characteristics of patients are presented in Table 1. No significant differences in demographic characteristics were seen between groups. One very preterm neonate (born in gestational week 31) was enrolled at a corrected age of 36 weeks without any complications. Thirteen patients had previous histories, comprising pneumothorax in 1, transient tachypnea of the newborn in 1, jaundice in 3, significant vomiting in 1, very preterm and very low birth weight infant in 1, late preterm and low birth weight infant in 3, congenital cystic adenomatoid malformation in 1, cleft lip, alveolus, and palate in 1, and congenital chylothorax in 1. No major adverse events occurred during treatment in either group. The primary outcome (healing rate after 2 weeks of treatment) and healing rate after 3 weeks of treatment are shown in Table 2. Healing rates counted patients that discontinued intervention or were lost to follow-up at earlier time points as failures. Efficacy rates of both treatments were almost identical after 3 weeks of treatment (Table 2), the lower value of the confidence interval was lower than the pre-specified non-inferiority margin of 10%, so the non-inferiority criterion was not statistically met. For reference, the Wald-type confidence interval was -15.35 to 4.35. Seven patients changed treatment after 2 weeks. Of these, in the SNC group, 2 patients underwent ligation and achieved cure after 3 weeks of treatment, and 2 patients changed to TSO treatment and achieved cured after 3 weeks of treatment. In the TSO group, 3 patients changed to SNC treatment, with one achieving cure after 3 weeks of treatment, and another after 4 weeks, while the remaining patient underwent ligation after 3 weeks and achieved cure after 4 weeks of treatment.

**Table 1:** Baseline characteristics of patients.

	SNC	TSO	Total
	(n = 104)	(n = 100)	(n = 204)
Male	59 (57%)	57 (57%)	116 (57%)
Age at diagnosis (days)	31 (7–39)	31 (8–38)	31 (7–39)
Gestational age (weeks)	39 (31–41)	39 (34–41)	39 (31–41)
Body weight at birth (g)	3027 (1092–3935)	3031 (1825–4212)	3029 (1092–4212)
Prehistory	5 (5%)	8 (8%)	13 (6%)
Moist discharge	99 (95%)	96 (96%)	195 (96%)
Bleeding	11 (11%)	9 (9%)	20 (10%)
Granulation	81 (78%)	78 (79%)	159 (78%)

Data are expressed as n (%) or median (inter-quartile range).

SNC, silver nitrate cauterization; TSO, Topical steroid ointment.

**Table 2:** Umbilical granuloma healing rate after 1, 2 and 3 weeks of treatment.

	Effective		Difference	95% Confidence Interval
	SNC	TSO		
	(n = 104)	(n = 100)		
**1 week**	57 (54.8%)	53 (53.0%)	-1.8%	(-15.5 to 12.1)
**2 weeks**	91 (87.5%)	82 (82.0%)	-5.5%	(-19.1 to 8.4)
**3 weeks**	94 (90.4%)	91 (91.0%)	0.6%	(-13.2 to 14.3)

Data are expressed as n or %. Healing rates counted patients that discontinued intervention or were lost to follow-up at earlier time points as failures.

SNC, silver nitrate cauterization; TSO, Topical steroid ointment.

## Discussion

This prospective, nonblinded, multicenter RCT compared the efficacies of SNC and TSO treatments in neonates with newly diagnosed UG. These results did not statistically confirm that the healing rate with TSO treatment was as good as that with SNC after 2-week treatment, but did show that healing rates of both treatments were very similar after 3-week treatment. These results suggest the TSO treatment as a potential first-line treatment for UG, owing to not only the favorable efficacy, but also the safety and simplicity.

To the best of our knowledge, this represents the largest RCT to compare the effects of SNC and TSO treatment on neonatal UG. The present study did not demonstrate the non-inferiority of the TSO group relative to the SNC group in terms of the primary endpoint. However, the efficacy of TSO was as good as that of SNC based on the results of the healing rate after 3 weeks of treatment although non-inferiority has not been met even at this stage. The two groups displayed similar demographic characteristics and endpoints were clearly defined in advance. On the other hand, healing rates of SNC and TSO treatment at 2 weeks (primary outcome) were 87.5% and 82.0%, respectively, and were lower than expected (95.0%). This may be why the non-inferiority criterion was not met. However, healing rates at 3 weeks were 90.4% and 91.0%, respectively, and were considered to be almost identical. Furthermore, healing rates at 3 weeks were compatible with a recent study by Brodsgaard et al that showed similar efficacies for topical clobetasol propionate cream (0.05%) and SNC in the treatment of UG. In that study, healing rates after 30 days of treatment with SNC and clobetasol propionate cream group were 96.6% (29/30) and 90.0% (27/30), respectively [11]. The present study enrolled a much larger patient cohort than that previous study, and demonstrated more clearly that TSO treatment should be considered as a feasible treatment option for neonatal UG.

TSO treatment appears superior to SNC with regard to safety and simplicity. The use of SNC is not without risk. In particular, iatrogenic periumbilical silver nitrate burns are still reported [3–6]. Although the high healing rate for UG and the low cost (less than $2 US per treatment) are advantages for SNC, the performance of this method justifiably carries a certain degree of concern among medical staff and parents. On the other hand, prolonged application of topical glucocorticosteroids can lead to skin atrophy, telangiectasia, hemorrhage, bacterial infections and even paradoxical inflammatory responses [14]. In addition, these agents can cause systemic adverse effects including Cushing’s syndrome and hypothalamic-pituitary-adrenal axis suppression, which should be avoided [15]. A complete review of the adverse effects of topical steroids, with guidelines and suggestions regarding their use, was reported by Hengge et al [14]. Children are more susceptible to the systemic adverse effects of steroids because of enhanced percutaneous absorption through their tender skin. Although the duration of treatment for neonatal UG, at a maximum of around 1 month, is short enough that such risks are not a concern [16], neonates have a larger ratio of body surface area to body weight than older children and should be followed carefully. Despite this, TSO can be conveniently applied at home by parents, safely and effectively, as long as they have the right information, thus reducing the burden on physicians in outpatient clinics. Moreover, TSO treatment is also inexpensive (less than $3 US per prescription). These advantages were stressed and confirmed in the present work, making TSO a good alternative in the treatment of UG in neonates.

In terms of the pathological mechanisms, glucocorticosteroids are known to suppress immune pathways in keratinocytes and to negatively affect the viability, maturation, and immune function of inflammatory cells [17, 18]. In addition, recent findings have shown that topical steroids may achieve anti-inflammatory effects and contribute to reduced numbers of fibroblasts [19, 20]. The lesion in UG is characterized by overgrowth of granulation tissue after umbilical cord separation. Brady et al showed histopathological findings for UG in a 2-month-old patient and demonstrated that UG predominately comprises granulation tissue consisting of fibroblasts, abundant small blood vessels, and mixed inflammatory infiltrate set in an edematous stroma [21]. TSO can reduce the progression of inflammatory process followed by a proliferative phase and this approach appears reasonable for neonatal UG.

This study has several limitations. First, treatment allocation was not blinded, since the differences in treatment drugs and tools could not be hidden from caregivers. In addition, we could not confirm that TSO treatment had been performed appropriately, since the application was carried out by the parents in their domiciles. Second, because we allowed each pediatrician to change to another treatment or ligate the UG after 2 weeks of treatment, two patients changed from SNC to TSO, three patients changed from TSO to SNC, and 4 SNC patients and 1 TSO patient underwent ligation, so the efficacy rate might thus have been assessed inaccurately. Third, we did not set a placebo group, as SNC is currently the standard of care for UG [1]. However, a dry cord has recently been shown to be non-inferior to the use of antiseptics just for care of the cord after birth in a developed country [22]. Therefore, even for UG, further study is needed to clarify the necessity for treatment. Moreover, two studies have already suggested that care with alcoholic wipes offers lower efficacy than SNC [5, 11]. Finally, we did not evaluate differences associated with the size or shape of the UG. A large UG may respond better to ligation, despite the good results observed and reported here with TSO. However, the present study represents the largest study to compare the efficacy of TSO treatment to that of SNC in patients with UG.

## Conclusions

This study did not establish the non-inferiority of TSO relative to SNC, possibly due to lower than expected healing rates, leading to a lack of power in this investigation. However, considering that SCN carries the risk of chemical burns when not used properly and that the overall efficacy of TSO treatment is similar to that of SNC, TSO treatment might offer a very good alternative in the treatment of neonatal UG. To confirm non-inferiority, a larger study is needed.

## Supporting information

S1 Fig CONSORT checklist. (PDF)

S2 Fig Trial protocol in Japanese. (DOC)

S3 Fig Trial protocol partially translated in English. (DOC)
