# Correction: Treatment with silver nitrate versus topical steroid treatment for umbilical granuloma: A non-inferiority randomized control trial

**DOI:** 10.1371/journal.pone.0218205

**Published:** 2019-06-11

**Authors:** Chikako Ogawa, Yoshiaki Sato, Chiyo Suzuki, Azusa Mano, Atsushi Tashiro, Takafumi Niwa, Sayako Hamazaki, Yoshihiro Tanahashi, Midori Suzumura, Satoshi Hayano, Masahiro Hayakawa, Takeshi Tsuji, Shin Hoshino, Yuichiro Sugiyama, Hiroyuki Kidokoro, Jun-ichi Kawada, Hideki Muramatsu, Akihiro Hirakawa, Masahiko Ando, Jun Natsume, Seiji Kojima

In the TSO treatment subsection of the Materials and methods section, the concentration of betamethasone valerate ointment is incorrect. The correct sentence is: Patients were applied with 0.12% betamethasone valerate ointment to the lesion twice a day after washing or bathing by parents.
