# Supplementary material for: Treatment with silver nitrate versus topical steroid treatment for umbilical granuloma: A non-inferiority randomized control trial
Source: PLoS One. 2018 Feb 13;13(2):e0192688. doi: 10.1371/journal.pone.0192688 (PMC5811027; doi:10.1371/journal.pone.0192688)
Supplement: S2 Fig — (DOC) [file pone.0192688.s002.doc]

**別記様式第２号の４**

臍肉芽腫に対する吉草酸ベタメタゾン軟膏の有用性について前方視的多施設共同研究

研究計画書（臨床介入研究）

研究代表者：

（小島勢二，小児科・教授，

466-8550　名古屋市昭和区鶴舞町６５　TEL.FAX:052-744-2994

E-mail:kojimas@med.nagoya-u.ac.jp）

研究事務局：（名古屋大学大学院医学研究科小児科

466-8550　名古屋市昭和区鶴舞町６５

TEL (052) 741-2111 (大代表) FAX (052) 744-2974）

平成25年3 月19日　作成　　　　　　　　　Ver.3

**研究計画書（臨床介入研究）**

**Ⅰ　課題名**

臍肉芽腫に対する吉草酸ベタメタゾン軟膏の有用性について前方視的多施設共同研究

A prospective study of the efficacy of Betamethasone valerate on umbilical granuloma

**Ⅱ　研究組織**

**１　研究責任者（小児科・教授　小島勢二）**

**２　研究分担者**

**（小児科・准教授　夏目淳，**

**周産母子センター・学内講師 佐藤義朗，**

**小児科大学院生 小川千香子，**

**半田市立半田病院・小児科医師　尾本梓，**

**中津川市民病院・小児科医師　多代篤史，**

**中津川市民病院・小児科医師　丹羽崇文，**

**名古屋第一赤十字病院・小児科医師　鈴木知世**

**名古屋大学医学部附属病院　先端医療・臨床研究支援センター・准教授　安藤昌彦**

**名古屋大学医学部附属病院　先端医療・臨床研究支援センター・講師　平川晃弘）**

**３　共同研究者**

**あいち小児保健医療総合センターアレルギー科・内科部長兼臨床検査部長・伊藤浩明**

**厚生連安城更生病院小児科・新生児科医長・竹本康二**

**春日井市民病院小児科・医長・星野　伸**

**名古屋大学医学部　保健学科　看護学専攻　教授　玉腰浩司**

**藤田保健衛生大学・公衆衛生学　教授　八谷 寛准**

**Ⅲ　研究等の概要**

臍肉芽腫と診断された児を対象とし、従来の治療法である硝酸銀焼しゃく療法と、新規治療である吉草酸ベタメタゾン軟膏治療群を比較し、その有効性を検討する。

**＜研究の背景・意義・目的＞**

臍肉芽腫はへその緒が脱落した後にできることのあるポリープ状の組織のことで、出血や細菌感染を伴うことがあり従来硝酸銀焼しゃく療法が行われてきた。しかし、硝酸銀による化学熱傷など有害な報告もある。学会ではステロイド軟膏の有用性について報告があるが、まとまった研究は行われていない。そのため今回両者を比較し臍炎や硝酸銀による化学熱傷などの有害事象がより少なく、肉芽の縮小率や湿潤の改善程度など評価することでより有効な治療法を検討することを目的とした。

**＜研究の対象＞**

１　対象者の選択基準・症例数　外来で視診にて臍肉芽腫と診断された生後1週間から5週間の新生児200例（症例数200例　治癒（湿潤無）率が両群とも95%以上、2群間の差が10%以内であれば、臨床的に差がないと考え、危険率5％、検出力80％、脱落率5％として算出）。

２　除外基準　臍炎の合併がある場合。臍部の外科処置をしたことがある場合。一週間以内の抗生剤全身投与を受けている場合。臍肉芽腫の治療をこれまでにすでに受けている場合。

３　採取する試料等：なし

□病理材料（対象臓器名　　　）　□生検材料（対象臓器名　　　）

□血液材料　□遊離細胞　□その他（　　　　　　）

・上記材料の採取期間　　西暦　　　年　　月〜　西暦　　　　年　　月

・採取の方法（侵襲性の有無）

・材料の数量等

・試料の保管場所（　研究室名まで記載　　　　　）

・新規採取試料か、既存試料か

４　使用する診療情報

妊娠分娩歴。

**＜研究期間＞**

実施承認日から　平成29年3月31日まで

**＜実施計画＞**

臍肉芽腫と診断された生後1週間から5週間の新生児を対象とする。臍肉芽腫治療法を硝酸銀（20%硝酸銀液焼しゃく療法）、吉草酸ベタメタゾン軟膏（1g中ベタメタゾン吉草酸エステル1.2mg含有）治療群に中央登録管理下での無作為割り付けにて無作為に分ける。硝酸銀治療群は従来の治療法通り、診察室で硝酸銀焼しゃく療法を行う。吉草酸ベタメタゾン軟膏治療群は自宅で1日2回塗布するよう指導する。両群とも1週間後、2週間後および3週間後に再診し視診にて治癒を判定する。2週間後で吉草酸ベタメタゾン軟膏治療群で改善に乏しい場合、従来の治療である硝酸銀焼しゃく療法を行う。吉草酸ベタメタゾン軟膏治療群で2週間後に効果を認めるが治癒まで至っていない場合には吉草酸ベタメタゾン軟膏治療を継続し、治療開始から3週間後に再診し、その時にも治癒していなければ従来の治療である硝酸銀焼しゃく療法を行う。視診は写真を用い客観的に評価できるようにする。治癒の評価としては肉芽の消失とする。写真は正面からと側面からの2方向で撮影を行い、肉芽のサイズ、湿潤の有無を評価項目として判定を行う。写真による客観的な判定は独立した判定者に治療群がマスクされた形で行う。撮影した写真は、匿名化されたまま厳重に保存され、診断、治療効果、予後判定のために使用される。主解析の統計手法は治癒割合の群間差の95％信頼区間下限が非劣勢マージンである10％を下回らないことを確認する。研究は名古屋大学大学院医学研究科小児科が中心となり多施設共同研究を行う。

**＜使用する研究費＞**

■運営交付金　　　□科学研究費　　　□厚生労働科学研究費

□その他公的研究費（　　　　　）□寄附金・研究助成金　□共同研究費（　　　　　　　　）、受託研究費（　　　　　　）　　□その他（　　　　　　）

**＜共同研究機関＞**

　本学を中心に共同研究を予定。共同研究機関への検体の提供等はありません。

**＜外部委託＞**

　予定なし。

**Ⅳ　研究等の実施場所**

**名古屋大学医学部附属病院**

**名古屋第一赤十字病院**

**中部労災病院**

**名古屋逓信病院**

**名古屋記念病院**

**津島市民病院**

**厚生連安城更生病院**

**岡崎市民病院**

**東海市民病院**

**厚生連豊田厚生病院**

**トヨタ記念病院**

**大垣市民病院**

**中津川市民病院**

**~~掛川市立総合病院~~**

**~~春日井市民病院~~**

**総合上飯田第一病院**

**公立陶生病院**

**Ⅴ　実施に際しての倫理的配慮について**

**Ⅴ―１＜研究等の対象とする個人の人権への対策＞**

個人情報は記号で匿名化して保存する。匿名で研究成果として発表されることがあり得ますが、個人を特定できる内容の発表にはしない。

**Ⅴ－２＜対象者を選ぶ方針・基準＞**

謝金等　　■無　　□有

**Ⅴ－３＜個人情報の取り扱いについて＞**

1. 個人情報管理施設が名古屋大学大学院医学系研究科・附属病院に…■該当する

□該当しない

2) 個人情報管理施設が名古屋大学大学院医学系研究科・附属病院に該当する場合

　個人情報管理者氏名：佐藤義朗

　資格（医師、臨床検査技師等）：医師

　個人情報管理補助者氏名：夏目　淳

　資格（医師、臨床検査技師等）：医師

（委嘱される場合、守秘義務が生じる）

3) 匿名化の有無………………………■匿名化する　　　□匿名化しない

4) 匿名化する場合……………………■連結可能匿名化　□連結不可能匿名化

5) 連結可能匿名化する場合は連結表の管理方法を具体的に記載：

　　鍵のついた保管庫に入れ管理する。

**Ⅴ－４＜対象者に理解を求め同意を得る方法＞**（下記の該当項目を選択する）

対象者各人に（**□1.書面のみ　 □2. 口頭のみ**■**3. 書面と口頭**）で説明した後

■A.　対象者の署名入りの同意書を保管する。

□B.　対象者の同意の署名が記された診療録を保管する。

□C.　対象者の同意の署名が記された調査票を保管する。

Ⅴ－５＜同意の撤回＞

この臨床研究への参加・不参加は両親の自由な意思によって決められ、また途中で撤回することも可能。不参加や参加を撤回してもそのために不利益を被ることはない。

Ⅴ－６＜対象者が未成年者の場合、成年者でも十分な判断力のない場合

又は病名に対する配慮が必要な場合などにおける対処方法。＞

□A　下記特例を対象にしない（対処方法の記載の必要がない）

■B　未成年者

□C　十分な判断力がない成年者

□D　意識のない成年者

□E　病名に対する配慮が必要な成年者

□F　その他（理由：）

具体的な対処方法：（下記の該当項目を選択。複数選択可。例外の場合は対処方法を具体的に記載。）

□インフォームド・アセントを取得する。

■保護者・代諾者の署名入りの同意書を保管する。

□保護者・代諾者の同意の署名が記された診療録を保管する。

□保護者・代諾者の同意の署名が記された調査票を保管する。

Ⅴ―７＜分析結果の開示＞

　　希望があれば研究に差支えない範囲で、同意書に署名した保護者・代諾者にこの研究計画の内容を開示する。

Ⅵ　期待される研究成果あるいは予測される利益

吉草酸ベタメタゾン軟膏治療群は、利益は化学熱傷のリスクが減り、本人の苦痛も少なくなると考えられる。不利益としては治癒までの期間が長くなることが考えられる。硝酸銀焼しゃく療法群は従来通りの治療を受けることができる。

Ⅶ　予測される危険と不利益とそれに対する配慮・補償

広く使われている薬剤の短期使用であり、薬剤そのものによる危険や不利益は考えにくい。治癒までの期間が長くなった場合は、硝酸銀焼しゃく療法に変更する。また、臍炎を合併した場合は、抗生剤治療を行う。

**Ⅶ－１＜研究等によって対象者に生じうる危険と不快に対する具体的配慮＞**

・治癒に時間がかかる。→1週間および2週間後で治療効果判定し、2週間後にも治癒していなければ従来の治療を行う。

**Ⅶ－２＜対象者に健康被害が生じた場合の補償の有無及び具体的な措置＞**

研究の種類を選択すること

■①介入を伴う研究であって、医薬品又は医療機器を用いた予防、診断又は治療方法に関するもの（対外診断を目的とした研究を除く）

「補償のための保険その他の必要な措置」について、事前に十分な説明を行い、対象者の同意を受けなければならない。

□②それ以外の介入を伴う研究（体外診断を目的とした研究を含む）

補償の有無を説明する必要がある。

□③観察研究にあたっては、試料等の採取が侵襲性を有する場合

補償のための保険等必要な措置の有無等を対象者に十分に説明する必要がある。

補償の種類を選択すること

□補償のための保険を設定した

■補償のための保険を設定する予定である（具体的内容：　　　　　　　　　　）

□保険以外の対処方法を講じた（具体的内容：　　　　　　　　　　　　　　　）

□補償がない旨を説明する

措置内容

□説明同意文書に補償のための保険等必要な措置について記載した

□補償のための保険等必要な措置を証明する文書を添付した

**Ⅷ　既存試料の利用**

注）既存試料：臨床研究計画書の作成時までに既に存在する資料又は臨床研究計画書の作成時以降に収集した資料であって収集の時点においては当該臨床研究に用いることを目的としていなかったものをいう。

１　既存試料の利用　□あり　■なし

**Ⅸ　研究期間終了後の試料の取扱い**

■　廃棄する

□　保存する

　　（保存するを選択した場合は下記について記載すること。）

　　ア　試料等の名称：

イ　試料等の保管場所：

ウ　試料等の管理責任者：

エ　被験者等から得た同意の内容（将来にわたって試料を解析する場合はその旨を対象者に説明し同意を得ること）：

**Ⅹ　備　考**

この研究において利益相反行為はありません。

**ⅩⅠ　参考文献**

1)Topical umbilical cord care at birth (Review); Cochrane Database Syst Rev. 2004(3)CD001057.

2)Higher Rate of Cord-Related Adverse Events in Neonates with Dry Umbilical Cord Care Compared to Chlorhexidine Powder; Neonatology. 2009_96(1)13-8.

3) Regime Versus Dry Cord Care

To Dye or Not to Dye: A Randomized, Clinical Trial of a Triple Dye/Alcohol Regime Versus Dry Cord Care; Pediatrics. 2003 Jan_111(1)15-20.

4) Role of antimicrobial applications to the umbilical cord in neonates to prevent bacterial colonization and infection: a review of the evidence; Pediatr Infect Dis J. 2003 Nov_22(11)996-1002.

5)Persistent umbilical discharge in infants and children; Ann Trop Paediatr. 2006 Jun_26(2)133-5.

6)Silver_nitrate_burns_following_umbilical_granuloma_treatment;10.1136/adc.2004.067918

7)臍肉芽腫治療の再検討-ステロイド軟膏の有用性について-;日本周産期・新生児医学会雑誌.47(2):347-347,2011.
