# Supplementary material for: Treatment with silver nitrate versus topical steroid treatment for umbilical granuloma: A non-inferiority randomized control trial
Source: PLoS One. 2018 Feb 13;13(2):e0192688. doi: 10.1371/journal.pone.0192688 (PMC5811027; doi:10.1371/journal.pone.0192688)
Supplement: S3 Fig — (DOC) [file pone.0192688.s003.doc]

A prospective multicenter randomized controlled study of the efficacy of betamethasone valerate on umbilical granuloma.

Name of lead principal investigator:

Seiji Kojima

Department of Pediatrics, Nagoya University Graduate School of Medicine

65 Tsurumai-cho Showa-ku, Nagoya 466-8550, Japan

Tel: +81-(0)52-744-2294

E-mail: kojimas@med.nagoya-u.ac.jp

Study Organization:

Department of Pediatrics, Nagoya University Graduate School of Medicine

65 Tsurumai-cho Showa-ku, Nagoya 466-8550, Japan

Tel: +81-(0)52-741-2111 FAX: +81-(0)52-744-2974

March 19^th^, 2013　Ver.3

Study protocol

**Ⅰ　Title of the study**

A prospective study of the efficacy of Betamethasone valerate on umbilical granuloma

**Ⅱ**Study Organization

**１　Name of lead principal investigator**

Department of Pediatrics・Professor Seiji Kojima

**２　Names of study partakers**

Jun Natsume

Yoshiaki Sato，

Chikako Ogawa，

Azusa Omoto，

Astushi Tashiro，

Takafumi Niwa，

Chiyo Suzuki

Masahiko Ando

Akihiro Hirakawa

**＜Patients of the study＞**

１　Inclusion criteria and sample size: Diagnosis of umbilical granuloma from clinical features; age, 1–5 weeks. 200 neonates. (In the sample size calculation for this non-inferiority trial, we assumed that the healing rate will be 95% for both treatment groups. The non-inferiority margin was set at 10%; that is, the aim of this non-inferiority trial is to demonstrate that topical steroid ointment treatment is no more than 10% worse than silver nitrate cauterization with respect to the healing rate. To this end, a sample size of 100 patients per group is required to achieve more than 80% power with a two-sided type 1 error of 5%.)

２　Exclusion criteria: Infection in the umbilicus; prior treatment of umbilical granuloma; prior systemic antibiotic treatment within 1 week; or prior surgical treatment of the umbilicus.

**＜Date of the study＞**

From: Date of protocol fixation - To: March 31, 2017

**＜Protocol＞**

Inclusion criteria are: 1) age, 1–5 weeks; and 2) diagnosis of umbilical granuloma from clinical features. Concealed randomization is conducted centrally through a computer-generated block randomization schedule. All patients meeting the inclusion criteria are randomized to receive either silver nitrate cauterization or topical steroid ointment treatment. Patients randomized as silver nitrate cauterization group are treated once a week by pediatricians using silver nitrate mounted on a clean stick applicator at a concentration of 20% in a hospital or clinic. Patients randomized as topical steroid ointment treatment group are applied with 0.05% betamethasone valerate ointment to the lesion twice a day by parents. All patients attend the outpatient clinic once a week. Changing or addition of another treatment is allowed if the lesion is not healed after two-week treatment. Evaluation is conducted using photography from two directions to judge the size of granulation and the presence of umbilical exudate blindly. Healing is defined as no clinical sign of umbilical exudate, bleeding or granulation. All photographs are linkable anonymized and only use to diagnose and judge the healing. The trial primary endpoint was the healing rate for the silver nitrate cauterization and topical steroid ointment groups after 2 weeks of treatment. The study is multicenter trial conducted centrally by the department of pediatrics at Nagoya University Graduate School of Medicine.
